# Machine learning model to study the rugby head impact in a laboratory setting

**DOI:** 10.1371/journal.pone.0305986

**Published:** 2025-01-06

**Authors:** Danyon Stitt, Natalia Kabaliuk, Nicole Spriggs, Stefan Henley, Keith Alexander, Nick Draper

**Affiliations:** 1 Department of Mechanical Engineering, University of Canterbury, Christchurch, Canterbury, New Zealand; 2 Department of Tourism, Sport, and Society, Lincoln University, Lincoln, Canterbury, New Zealand; 3 Faculty of Health, University of Canterbury, Christchurch, Canterbury, New Zealand; 4 Sports Health and Rehabilitation Research Center (SHARRC), University of Canterbury, Christchurch, Canterbury, New Zealand; KPC Medical College and Hospital, INDIA

## Abstract

The incidence of head impacts in rugby has been a growing concern for player safety. While rugby headgear shows potential to mitigate head impact intensity during laboratory simulations, evaluating its on-field effectiveness is challenging. Current rugby-specific laboratory testing methods may not represent on-field conditions. This study aimed to create a machine-learning model capable of matching head impacts recorded via wearable sensors to the nearest match in a pre-existing library of laboratory-simulated head impacts for further investigation. Separate random forest models were trained, and optimised, on a training dataset of laboratory head impact data to predict the impact location, impact surface angle, neck inclusion, and drop height of a given laboratory head impact. The models achieved hold-out test set accuracies of 0.996, 1.0, 0.998, and 0.96 for the impact location, neck inclusion, impact surface angle, and drop height respectively. When applied to a male and female youth rugby head impact dataset, most impacts were classified as being to the side or rear of the head, with very few at the front of the head. Nearly 80% were more similar to laboratory impacts that included the neck with an impact surface angled at 30 or 45° with just under 20% being aligned with impacts onto a flat impact surface, and most were classified as low drop height impacts (7.5-30cm). Further analysis of the time series kinematics and spatial brain strain resulting from impact is required to align the laboratory head impact testing with the on-field conditions.

## Introduction

Head impact exposure within sports has been linked to adverse mental and physical health outcomes, even without a clinically diagnosed traumatic brain injury (TBI) [1–3]. Concussion is especially prevalent in rugby [4–6], where players experience an average of 14–52 significant head impacts per game [7–9]. Rates of mTBI in rugby vary between cohort, country, and level of play with studies reporting between 0.4 and 46 mTBIs per 1000 player hours [10–16] making it one of the most common injuries in the sport [4–6, 17]. In terms of protective equipment, rugby players show mixed opinions and beliefs, especially regarding the rugby-specific soft-shelled headgear. Studies investigating the rates of headgear usage report 2–27% of players wearing rugby headgear, with most studies reporting rates between 2–15% [18–23]. Those studies reporting the highest rates of headgear usage were specific to American and Canadian rugby players [21, 23]. Studies that also investigated player’s attitudes towards the protective performance of rugby headgear found that up to 62% of players believed headgear would protect from concussive injury [21–23], while the one study reporting coach’s attitudes reported that 33% of them held the same belief [23]. Many of the studies report a disconnect between awareness and attitudes towards concussion, and player behaviour, with many players ignoring return-to-play guidelines, deliberately influencing concussion tests, or simply disregarding the long-term effects of concussive injuries [24–27].

This divide is not unfounded. The scientific community lacks a clear consensus on whether soft-shelled rugby headgear could reduce the risk of concussive injury. Nearly all investigations in a laboratory setting find that the presence of rugby headgear significantly reduces the peak linear and rotational head impact accelerations associated with concussive injury [28–33]. Unfortunately, the results are unclear on the field. Most studies of the efficacy of headgear to reduce concussive injury risk during gameplay have found no significant difference in injury rates between those wearing and those not wearing headgear [34–38], with only one study finding a lower concussive injury rate in the headgear wearing cohort [39]. As discussed in a review article on soft-shelled rugby headgear [40], variability in the research design and concussion definition used within these articles complicates any direct comparison. The same review article reported that many of these studies did not consider confounding variables, such as the psychological effects of headgear use and specific risk-taking behaviour of those wearing headgear versus those without. A study of rugby players’ tendency to engage in aggressive play found that those who believed headgear prevented concussion were, on average, 4 times more likely to play in a more aggressive form than those who believed headgear did not prevent concussions [21].

Until recently, strict limits on the development of protective headgear were imposed by World Rugby [41]. The requirements for headgear design have been relaxed in World Rugby’s Law 4 trial assessment [42], bringing forth a new generation of headgear designs such as the Npro and Gamebreaker soft-shelled headgear. Specifically, the limit on material density (previously limited to ≤45*kg*/*m*^3^) and prohibition of sandwich construction were lifted to promote such innovations in headgear that may reduce head impact intensity in rugby. Despite this change, the standard methods used to simulate laboratory rugby head impacts remain unchanged. The most recent World Rugby standard requires headgear, fitted to a steel headform conforming to EN960 [43], to be dropped onto a steel impact surface from heights of 15–60*cm* with no clear criteria for interpreting the resulting impact kinematics. Most laboratory studies aiming to recreate rugby-specific head impacts for assessing impact attenuation of headgear, however, use a drop test method more closely aligned with the NOCSAE standard for evaluating American football helmets [28, 29, 31, 32, 44]. Although the NOCSAE standards cover a range of head impact testing methods, such as pneumatic ram and pendulum impacts, the drop testing method is the most commonly used for rugby headgear testing. The NOCSAE drop test standard requires a NOCSAE-specific headform to be dropped onto a modular elastomer programmer (MEP) pad at a range of impact velocities corresponding to free-fall drop heights of about 15 − 90*cm* [44]. However, many laboratory headgear studies use the Hybrid III (HIII) headform instead of the NOCSAE headform.

mTBI are believed to arise from a rapid change in motion of the head, specifically a change in rotational motion, causing excess strain on, and subsequent damage to, the axons in the brain. Such damage is commonly related to kinematic measures of head motion such as the peak linear and rotational acceleration (PLA and PRA) and the peak rotational velocity (PRV) as they are easily, and non-invasively, measured with accelerometers and instrumented mouthguards. Studies reporting the differences in the head impact kinematics across a range of drop test conditions have found seemingly minor changes to have a significant effect [45–48]. The head mass has been reported to decrease the peak linear acceleration (PLA) for the same impact velocity during drop testing [45], while headform shape was found to significantly affect the peak rotational acceleration (PRA) and velocity (PRV) during matched oblique drop tests, with the NOCSAE headform producing 20—30% lower values than the HIII headform [46]. Oeur and Hoshizaki found that the peak linear and rotational accelerations decreased as the compliance of the impact surface increased for matched drop test impacts [47]. A study by Stitt et al. found substantial differences in the peak accelerations, the ratios of the peak kinematics, and the kinematic time-series shape between variations in the drop test method [48]. Similar to Oeur and Hoshizaki, Stitt et al. found that increasing the impact surface compliance reduced the PLA and PRA of an impact and increased the duration of the linear acceleration peak for matched impacts. Introducing a HIII neckform was found to increase the duration of the peak rotational velocity, and alter the shape of the rotational acceleration temporal profile, but had little effect on the peak acceleration and velocity values. Altering the angle of the impact surface was found to have minimal effect on the peak kinematic values or the shapes of the traces.

Unfortunately, there is limited information regarding the impact mitigation of rugby headgear across different impact conditions. There has, however, been a significant amount of research investigating the impact attenuation of ice hockey headgear using methods that may better reflect on field conditions than standard drop tests. Clark and colleagues measured the kinematic and brain strain attenuation of ice hockey helmets for different impact events in ice hockey [49, 50], finding that the helmets had a diminishing effect on impact duration, peak accelerations, and peak maximal principal strain as the impact surface became more compliant compared to unhelmeted conditions. In both studies, the authors reported this to arise from the materials in the ice hockey helmets being stiffer than those of the impact surface. As a result, the helmet materials do not compress enough to absorb the impact energy, thereby minimizing impact attenuation. A study by de Grau and colleagues [51] also found that the reductions in peak kinematics and brain strain through the use of an ice hockey helmet were higher during low compliance impacts compared to high compliance impact conditions. A further study by Haid and colleagues performed drop tests onto surfaces with varying compliance, comparing the impact attenuation of ice hockey helmets [52]. When increasing impact surface compliance, the difference between helmeted and unhelmeted impacts decreased until a fitted helmet made no measurable difference to the peak accelerations.

Despite this work, there has been no direct comparison of the head impact conditions and kinematics that exist during rugby gameplay and training to those simulated in the laboratory. A method for simulating rugby-specific head impacts in the laboratory that aims to match the resulting kinematics that exist on the field as closely as possible would benefit the understanding of head impact biomechanics, head and brain injury biomechanics, and the development of protective headgear. Therefore, this study aimed to create a machine-learning model capable of matching head impacts recorded via wearable sensors to the nearest match in a pre-existing library of laboratory-simulated head impacts for further investigation.

## Materials and methods

### Data collection

**Fig 1:**
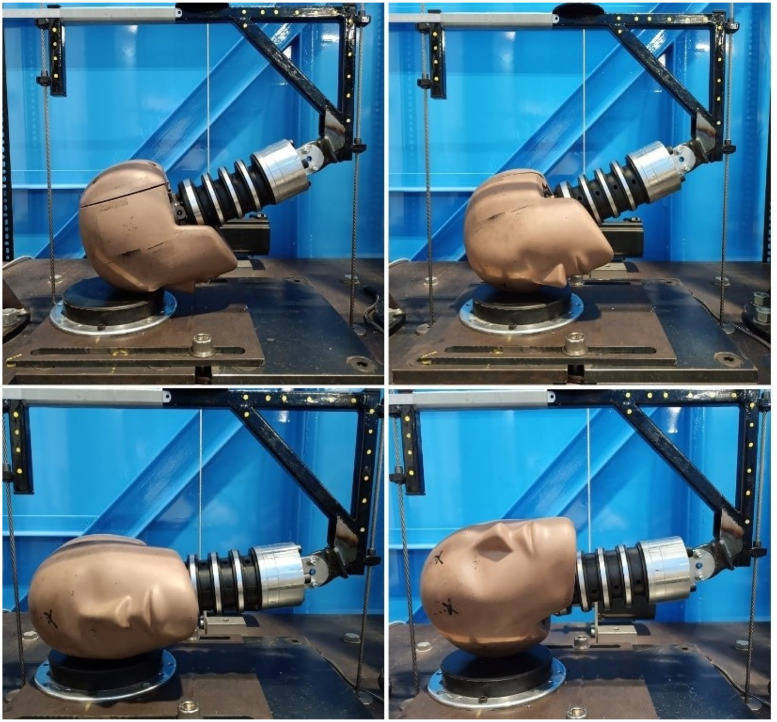
Laboratory impact locations onto a 0°MEP pad impact surface. From top left to bottom right: Forehead, Front boss, Side, Rear-rear boss. Image from [29], used under (CCAL) CC BY 4.0, copied from the original.

**Fig 2:**
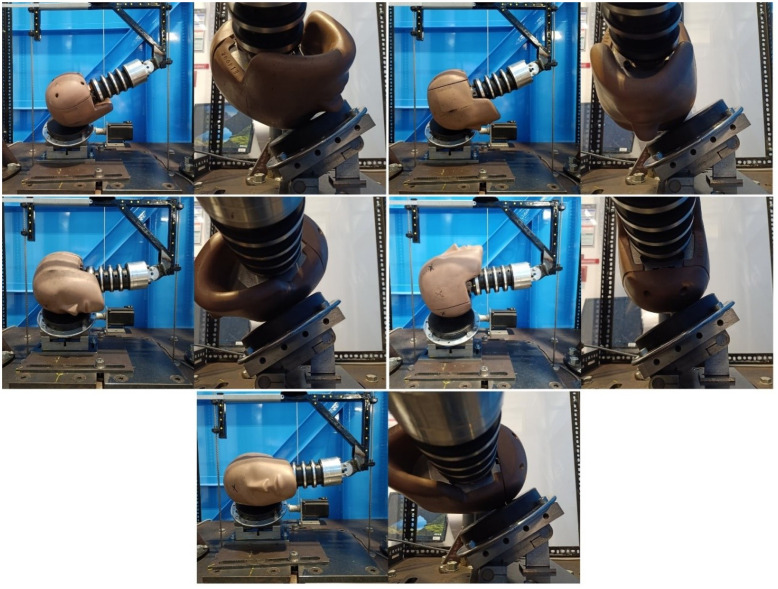
Laboratory impact locations onto a 30° and 45°MEP pad impact surface. From top left to bottom: Forehead, Front boss, Side, Rear-rear boss, Side-rear boss. Image from [29], used under (CCAL) CC BY 4.0, copied from the original.

All laboratory impacts were carried out on a twin wire guided drop test rig using an HIII headform instrumented with four triaxial accelerometers (Analog Devices ADXL377, 20,000*Hz*, range: ±200*g*, sensitivity: 6.5 *mV/g*) arranged into a nine accelerometer package (NAP) [53] with three redundant sensing axes. This allowed linear and rotational accelerations, and rotational velocity, to be measured and calculated. Accelerometers were positioned at the center of mass of the headform, and then orthogonally in the x, y, and z directions separated by between 52 and 75*mm*. Three variations of the drop test method were included based on previous work by Stitt et al. and Draper et al. The first used an HIII head and a 1-inch MEP pad impact surface, with no neck involved [28, 48]. The second and third drop test variations used were taken from the same authors’ study of rugby headgear [29]. Using the same HIII head and neck, and 1-inch MEP pad impact surface, drop tests were carried out with the impact surface angled at 0°, 30°, and 45° relative to the test rig base. Impacts onto the flat impact surface were carried out across four impact locations: forehead, front boss, side, and rear boss (labelled rear-rear boss), as shown in Fig 1. Impacts onto the 30° and 45° impact surfaces also included a fifth impact location labelled side-rear boss, shown in Fig 2. Impacts both with and without headgear were included in the dataset, resulting in 1806 individual impacts spread across all drop test conditions.

Field head impact data were recorded from two club rugby union teams: one male and one female, both from Christchurch, New Zealand, over the 2022 and 2023 seasons of play for males and over the 2022 season for females. Player recruitment began in March 2022 and ended in April 2023. Ethics approval was attained from the Human Ethics Committee of the University of Canterbury, reference: HEC 2021/26. Written informed consent was received from all players, and their parents, prior to the study. Players were followed across their club and school games and training. The age range of the players was 14–16 years old for the males and 13–17 years old for the females. Head impact kinematics were measured using the HitIQ nexus A9 mouthguards, previously validated under laboratory conditions by Stitt et al. [54]. The threshold for recording data was 8*g*. Retrospective video verification identified positive direct and indirect head acceleration events. The field head impact dataset comprised 440 male and 239 female video-verified, direct head impacts. A direct head impact was defined as a head acceleration event arising from a direct impact between an external body and a player’s head. Field head impact locations were also approximated during the video verification process with only impacts to the forehead, front boss, side, and rear regions of the head considered for this study to match the laboratory dataset.

### Data processing and feature extraction

Laboratory accelerometer data were recorded as raw voltages by a LABVIEW system using a National Instruments cDAQ 9171 as the interface between the sensors and LABVIEW. Raw voltages were converted into accelerations using Python 3.8. All laboratory kinematic data were filtered using a Butterworth low-pass filter with a cutoff frequency of 300*Hz*. The order and cutoff frequency of the low-pass filter were chosen to match that of the HitIQ in house post processing of the mouthguard head acceleration data. A set of 50 kinematic single-value features were extracted from each head impact and used to develop the kinematic feature set for the laboratory and field datasets. The feature set included: Peak resultant accelerations and velocities, the change in *x*, *y*, *z* accelerations and velocities, peak *x*, *y*, *z* accelerations and velocities, ratios of peak kinematics, injury metrics, ratios of injury metrics, and the duration of resultant kinematic peaks. The change in directional kinematics was calculated as the maximum value minus the minimum value and the injury metrics included were the HIC and RIC [55]. The duration of peak kinematics was calculated at 30% of the peak value as this was found to be the most reliable when used on field data where the rotational kinematics did not always return to zero post-peak. These features were chosen as they are easily interpreted in terms of a physical mechanism. A summary of these features can be found in S1 Table.

To describe the conditions required to carry out an impact on the drop test rig, only four parameters need to be known. These are the impact location, the impact surface angle, whether or not to include a neck, and the drop height. In addition, the impact surface characteristics would also need to be known, however, as there was only one impact surface involved in the laboratory dataset, this analysis was not carried out. Based on a previous investigation of laboratory impact kinematics under different conditions [48], it was assumed that different features would best predict the different drop test parameters. Based on this, separate models were built to predict each of the four parameters associated with drop tests.

### Classifier selection

Laboratory data was first split into training and testing sets of 70 and 30% of the entire dataset respectively using the stratified train test split function from the sklearn library. Stratification was based on the target class (e.g. the specific location for the impact locations). A k nearest neighbour, a logistic regression, and a random forest model were all assessed for predicting each target parameter. These classification algorithms were chosen due to their interpretability, ease of implementation in Python using the sklearn library, and use within the literature [56–58]. Each algorithm was assessed using the grid search function in sklearn where the hyperparameters were also tuned at the same time. Within this search, the best feature selection method was found between the f-classification and the mutual information scores. Other feature selection methods such as permutation feature importance were not considered due to the presence of correlated features within the features set. The performance of the algorithms was assessed using the average accuracy from 10-fold cross-validation of the training data. Other algorithms such as support vector machines, and gradient boosting trees were excluded as they were much more computationally intensive to run, and preliminary testing showed little to no improvement over the algorithms selected for testing, which were also much faster learners. The hyperparameters that were tuned were the number of features (from 1 to 20), the number of neighbours (for the KNN from 5 to 15), the penalty for the logistic regression (between l2 and none) and the inverse regularisation strength, and the number of estimators (from 50 to 200) and max depth (from 1 to 10) for the random forest model. All other hyperparameters were left as their default within the sklearn library. This process was carried out with separate classifiers for each drop test parameter.

### Model assessment and field condition prediction

Each model built using the optimal hyperparameters was assessed on the hold-out test set to ensure the best cross-validation accuracy did not come from overfitting to the training data. The accuracy was used to evaluate the different models instead of receiver operating curves and the corresponding AUROC, as most of the classification problems were multiclass and not binary. Finally, each of the models were used together to predict the respective drop test parameter which were combined for each impact in the hold-out test set to create the full set of impact parameters associated with each drop test impact. The accuracy of this total prediction was found by taking the percentage of fully correct predictions from the total number of predictions made. The final model was then used to predict the drop-test parameters for all field impacts. The impact location predictions were compared to the video approximated impact locations using a confusion matrix style analysis. This was mostly an early exploratory analysis to see what the most common impact parameters were and the differences, if any, between the males and females. The distribution of each predicted condition within each drop test parameter was subsequently found for the field head impact dataset.

## Results

The random forest models gave the highest accuracy across most drop-test parameters. This was followed by the k-nearest neighbours models and then the logistic regression models. It should be noted that all models gave the same cross-validation accuracy when predicting the neck status, however, for continuity with the other models the random forest model was also chosen for predicting this parameter. The cross-validation and test set accuracies, along with the optimal hyperparameters found during the grid search, for the random forest model are shown in Table 1. When predicting the impact location, the random forest model had an average cross-validation accuracy of 0.998, followed by the KNN model with 0.992, and the logistic regression model with an accuracy of 0.983. All models resulted in the same average cross-validation accuracy of 0.998 for the neck status. The impact surface angle was predicted with an average cross-validation accuracy of 0.995 with the random forest model followed by the KNN model at 0.994, then the logistic regression at 0.888. Finally, the drop height was predicted with an average accuracy of 0.993 with the random forest model, 0.991 with the KNN model, and 0.990 with the logistic regression model. The best feature selection method was found to be the mutual information classification method across all drop test parameters for the random forest model. A detailed classification report showing the class-wise precision, recall, and F1-score can be foun in the S2–S5 Tables.

**Table 1:** Mean (SD) cross-validation accuracy, the test set accuracy, and the optimal hyperparameters used for each random forest model.

Drop test parameter	CV accuracy	Test set accuracy	Features	Max depth	Estimators
**Impact location**	0.998 (0.004)	0.996	10	7	90
**Neck status**	0.998 (0.005)	1.0	1	1	50
**Impact surface angle**	0.995 (0.006)	0.998	7	10	200
**Drop height**	0.993 (0.007)	0.96	6	9	120

The impact location required 10 features with 90 estimators and a maximum tree depth of 7 nodes. The features that made up the model were the *x* and *y* peak linear acceleration and rotational velocity, the relative directional peak linear and rotational accelerations and velocities, along with the vector direction of the peak kinematics (Table 2). The neck status model only required a single feature, 50 estimators, and a single decision node. The single feature required for prediction was the duration of the rotational velocity peak at 30% of the peak value. The impact surface angle model required 7 features, with 200 estimators, and a max tree depth of 10 nodes. The features involved were the directional peak rotational velocities and the relative directional linear and rotational peak kinematics. Drop height was best predicted with the resultant peak linear acceleration and rotational velocity, followed by the change in linear velocity, the HIC, and the RIC. This model required 6 features with 120 estimators and a max tree depth of 9 nodes.

**Table 2:** Features used for each random forest model.

Impact location	Neck status	Impact surface angle	Drop height
PLA_*x*_	PRV duration	PRV_*x*_	PLA
PRV_*y*_		PRV_*z*_	PRV
Relative PLA_*x*_		Relative PLA_*y*_	Delta LV
Relative PLA_*y*_		Relative PRA_*x*_	Delta LV_*x*_
Relative PRA_*y*_		Relative PRA_*y*_	HIC
Relative PRV_*y*_		Relative PRA_*z*_	RIC
Relative PRV_*z*_		Relative PRV_*x*_	
PLA vector direction in xz plane			
PRA vector direction in xz plane			
PRV vector direction in xz plane			

When all four models were combined to predict the full set of drop test parameters for each impact in the hold out test set, the test set accuracy was 0.983. The incorrect predictions are compared to the actual impact parameters in Table 3. In most cases, only one of the drop test parameters was incorrectly classified, the exception being a forehead impact onto the 30°MEP pad which had the impact location and the impact surface angle misclassified. The algorithm correctly predicted all but three of the impact location conditions, with the algorithm incorrectly classifying two forehead impacts as front boss impacts and one front boss impact as a forehead impact. Four of the impacts had the impact surface angle incorrectly classified. These misclassifications were between 0° and 30° or 30° and 45°. No impacts onto the 0 MEP pad were classified as impacts ponto the 45°MEP pad, or vice versa. There were three incorrect predictions by the drop height model. Two were misclassified as the adjacent drop height and a third 30cm impact was classified as a 15cm impact. There were no recorded incorrect predictions of the neck status within the hold-out test set.

**Table 3:** Incorrect predictions of the entire set of drop test parameters for each impact in the hold out test set. The incorrect prediction, and the correct counterpart, is highlighted in bold font.

Prediction	True labels
Side neck 45°**15cm**	Side neck 45°**30cm**
**Forehead** neck 45°60cm	**Front boss** neck 45°60cm
Rear-rear boss no neck 0 **15cm**	Rear-rear boss no neck 0 **7.5cm**
Side-rear boss neck 45°**60cm**	Side-rear boss neck 45°**45cm**
Rear-rear boss no neck **30**° 22.5cm	Rear-rear boss no neck **0**° 22.5cm
Side-rear boss neck **0**° 45cm	Side-rear boss neck **30**° 45cm
**Front boss** neck **0**° 45cm	**Forehead** neck **30**° 45cm
**Front boss** neck 30°45cm	**Forehead** neck 30°45cm
Side-rear boss neck **45**° 15cm	Side-rear boss neck **30**° 15cm

### Field data predictions

**Fig 3:**
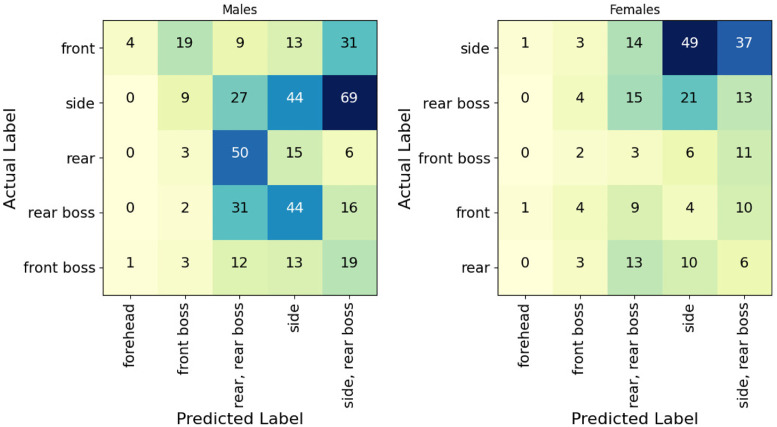
Predicted impact locations of male (right) and female (left) head impact data compared to the impact location identified during the video verification process.

The predicted impact location of each of the field head impacts compared to the approximated impact location from video verification is shown in Fig 3. While the predicted impact location agreed with the video approximated impact location for some impact locations, there were a large number of discrepancies. Specifically, male head impacts labelled as forehead impacts were commonly classified as front boss or side-rear boss impacts. Male head impacts that were labelled as front boss impacts were most commonly classified as an imp[act to the side, or rear of the head. Similar results were seen in the female head impact location predictions. It was assumed that an impact to the front boss or the rear boss could easily have been misidentified as an impact to the side of the head, and vice versa, during video verification. Discrepancies such as these, therefore, were not believed to be an issue. Male and female impacts to the side of the head were commonly predicted as such, while many were classified as side-rear boss impacts. A similar observation was made with impacts labelled as rear or rear boss impacts.

**Fig 4:**
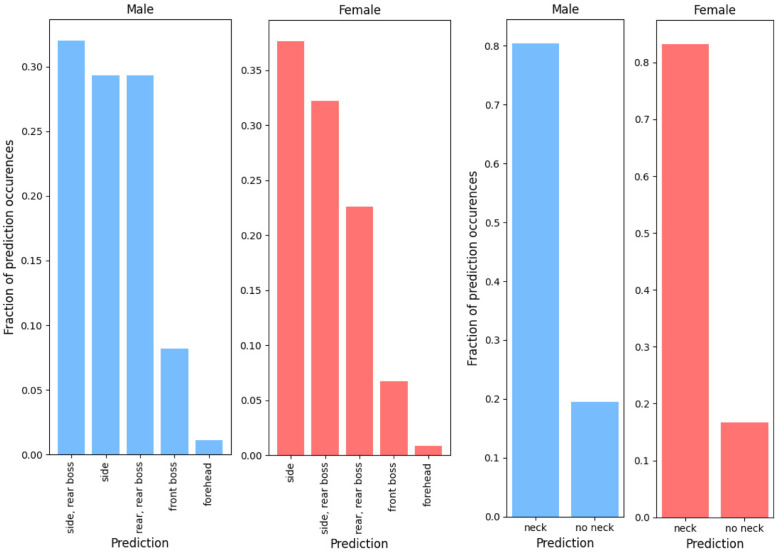
Fraction of each Impact location (right) and neck status (left) prediction classes within the field data as predicted by the random forest models. The blue shows males, red shows females.

**Fig 5:**
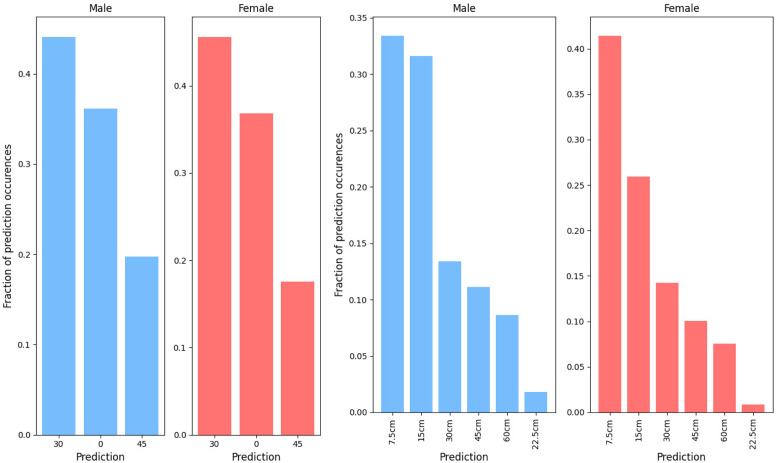
Fraction of each impact surface angle (right) and drop height (left) prediction classes within the field data as predicted by the random forest models. The blue shows males, red shows females.

Figs 4 and 5 show the fraction of each predicted drop test parameter within the field data as predicted by the random forest models. The most common impact location was the side-rear boss for males, followed by the side, rear-rear boss, the front boss, and the forehead. For females the most commonly predicted impact location was the side of the head, followed by the side-rear boss, rear-rear boss, the front boss, and the forehead. Forehead impacts were by far the least frequent. Minimal differences were seen between males and females. Both male and female field data were predicted to require inclusion of the neck in around 80% of cases, with the remaining impacts being more similar to those carried out with no neck involved (Fig 4). In both male and female groups, the most commonly predicted impact surface angle was 30°, followed by 0°, and finally 45°, comprising around 45%, 35%, and 20% in each group respectively. Drop heights, again, showed a strong similarity between the males and females with around 33% of male and 40% of female head impacts being predicted as 7.55cm drops, followed by 15cm, 30cm, 45cm, and 60cm. Only a few impacts were predicted as 22.5cm drops.

## Discussion

Each random forest model performed exceedingly well during cross-validation and hold-out testing on the laboratory data. As assumed, each drop test parameter was predicted by distinctively different kinematic features, justifying the choice of individual models for predicting each drop test parameter. The impact location prediction model used the *x* and *y* direction peak linear acceleration and rotational velocity, as well as the relative directional peak kinematics, both linear and rotational. The vector directions of the linear and rotational acceleration were also included, however including these features only resulted in marginal improvements in the model’s accuracy. These kinematics are influenced by the direction of the impact, which appeared to be strongly indicative of the location of the impact. During evaluation on the hold-out test set, the impact location model incorrectly identified two forehead impacts as front boss impacts and one front boss impact as a forehead impact. These, however, were relatively minor misclassifications as the front boss and forehead impact locations are next to each other. A more serious misclassification would be predicting a forehead impact as a rear boss impact, however such a prediction error was not observed. The impact surface angle classifier, while also using the peak and directional kinematics, favoured the rotational velocity and acceleration kinematics. This was interesting as a previous investigation of the effect of impact surface angle on the kinematics resulting from drop test head impacts found there to be almost no difference in the resultant peak rotational velocity between impacts onto a flat surface and one angled at 45° [48]. It should be noted that out of all the classifiers, this model required the highest maximum tree depth and number of estimators, suggesting there may be less distinction between impacts on the flat and those onto an angled surface.

The model classifying whether a neckform was used in the drop test was the most accurate classifier created in this study. This may have been due to the binary classification problem or due to a significant effect of the neck on the drop test kinematics. The model only required one feature and a single decision node to achieve an accuracy of 0.998 during cross-validation and a hold-out test set accuracy of 1.0. This feature was the duration (in *ms*) of the resultant rotational velocity peak. Again, this makes sense as the same comparison of drop test parameters on the resulting head impact kinematics found the main effect of including a neck was an increase in the duration of the rotational velocity peak while leaving the other kinematics relatively unchanged, including peak resultant accelerations and velocities [48].

While the drop height could theoretically be estimated using only the change in resultant linear velocity, the predictive model incorporated kinematics that seemed unintuitive and likely resulted in increased prediction accuracy compared to the linear velocity alone. This model required 6 features centred around peak resultant kinematics, the change in linear velocity, and the injury criteria. Similar to the impact location classifier, the drop height classifier incorrectly identified the drop heights of three impacts as 15, 15, and 60cm in the hold-out test set. The true drop heights of these impacts were close to those predicted, with the first being 30cm, the second being 7.5cm, and the third being 45cm. It is important to note that drop height is an ordinal variable, meaning both regression and classification could be used. Drop height prediction performance may increase by using a regression algorithm, however, this was not investigated due to the high accuracy of the classification algorithm. Ordinal regression problems may benefit from an implementation of ordinal logistic regression. Again, due to the high classification accuracy, this was not investigated.

Discrepancies between the predicted and video-verified impact locations were observed when looking at the predicted labels of the field head impact data. Such differences could easily be due to a mistake during video verification. Many recordings of the impacts were partially obstructed by other players, thus, attaining a confident impact location description of the impact location may not have been possible for every field head impact. Despite this, many of the predicted impact locations matched those of the video verification. Common discrepancies were predicting an impact labelled as a rear impact via video verification as a rear-rear boss, side-rear boss, or side impact, and vice versa. In both male and female datasets, video-labelled front boss impacts were most commonly predicted to be side-rear boss, side, or rear-rear boss impacts. There may be a difference in the injury potential between impacts to the front, rear, and side of the head, which is important for interpreting the severity of the misclassifications of the impact location model in this study. For example, misclassifying a forehead impact as a front boss impact would not be as detrimental as misclassifying a forehead impact as a side-rear boss impact. Additionally, if these models are to be used to investigate the impact mitigation of rugby headgear, it is important to note that there are significant differences in the impact mitigation of rugby headgear between impacts to the front, side, and rear of the head [28, 29]. This should also be considered when evaluating the severity of the model misclassifications. This is also true for the drop height, where incorrectly classifying a 15cm impact as a 30cm impact would not be as bad as classifying the same impact as a 45 or 60cm impact.

Both male and female head impact data sets shared similar proportions of each predicted condition within each of the drop test parameters. For the impact location, the side and rear of the head were the most commonly predicted locations. The front boss and forehead locations showed dramatically fewer prediction occurrences than the other impact locations. This could be due to the way that rugby players are taught to tackle or the specific biomechanics of tackling where players go in with their shoulder, leaving the side of the head open to impact. Most (around 80%) of the field impacts were predicted to be more similar to impacts with the neckform involved than without. This likely means that, in general, longer duration rotational velocity peaks are present in rugby head impacts compared to those in the laboratory. This could arise from the relatively unconstrained nature of the human head and neck during gameplay compared to that of the HIII head and neck in the laboratory. During and following a head impact, the head, neck, and body of a person are free to move and rotate. This may create rotational velocity data that has a large peak duration compared to the laboratory where the head and neck are fixed to a drop frame, which in turn is guided by tightened steel wires. This setup does not have the same freedom to move and rotate following an impact that likely exists within a real-life scenario.

The most commonly predicted impact surface angle for the male and female field head impact data was the 30°impact surface angle. This was followed by the 0°impact surface and then the 45°impact surface angle. The proportions of each of these within the male and female datasets were nearly identical. Physically, this implied that most of the head impacts recorded were due to a glancing blow to the head, rather than an impact where the line of action of the force was travelling near parallel to the direction of head travel. Additionally, from the video verification process, it was seen that most impacts were glancing blows to the head with either another player’s body or the ground. Such impacts were rarely a direct head-on collision as would be simulated in the laboratory with an impact onto a flat impact surface.

The most commonly predicted drop height was 7.5cm for both males and females, followed by the 15cm and 30cm drop heights. This indicated that most of the impacts experienced by the study cohort were at lower impact velocities and, by extension, severity. While few of these impacts would have a change in linear velocity matching that of the predicted drop height, these results do indicate the relative proportion of drop heights that reflect on-field conditions. For recreating specific impacts in the laboratory it may be more useful to find the change in linear velocity directly from the mouthguard kinematic data and calculate the drop height associated with this velocity change.

The classification models developed in this study provide a solid basis for exploring the relationship of laboratory-simulated head impacts to those measured during youth rugby union. There are, however, some significant limitations. Firstly, this analysis does not directly compare the time series traces. Instead, the models only predict the closest matching impact within our library of head impact simulations based on single-value kinematics. Such a comparison would be necessary to fully understand the relationship between rugby head impact conditions and the laboratory simulations of said impacts. Additionally, these classifiers do not take into account any brain strain data which may be one of the most important features to preserve between the field and the laboratory. Secondly, the predictive classifiers are only able to make predictions based on what has been generated for the library of laboratory head impacts. Many of the field impacts may be better reflected by other impact surfaces or head sizes or even by a different method of testing such as a pneumatic ram or pendulum impacts. Finally, the field dataset should be extended. This would allow the field head impact dataset to capture a broader range of on-field head impact conditions, allowing the predictive models developed here to be assessed under a greater range of head impact conditions.

## Conclusion

This study provides a method of matching impacts recorded during youth rugby to those in a pre-existing library of head impacts. This can be carried out for any sport of interest and any head impact simulation method of interest. Such a method may aid in the understanding of the role of protective equipment during specific head impacts. However, the library of drop test simulations used in this study did not include a variety of impact surfaces. Rugby head impacts likely cover a range of impact surface stiffnesses. Extending the library of laboratory head impact conditions would be necessary to correctly predict, and subsequently recreate, a given field head impact.

## Supporting information

S1 Table Features, along with their explanation, extracted from each impact. (JPG)

S2 Table Classification report for the random forest impact location classifier. (JPG)

S3 Table Classification report for the random forest neck status classifier. (JPG)

S4 Table Classification report for the random forest impact surface angle classifier. (JPG)

S5 Table Classification report for the random forest drop height classifier. (JPG)
