# Supplementary figures and images for: Machine learning model to study the rugby head impact in a laboratory setting
Source: PLoS One. 2025 Jan 6;20(1):e0305986. doi: 10.1371/journal.pone.0305986 (PMC11703033; doi:10.1371/journal.pone.0305986)

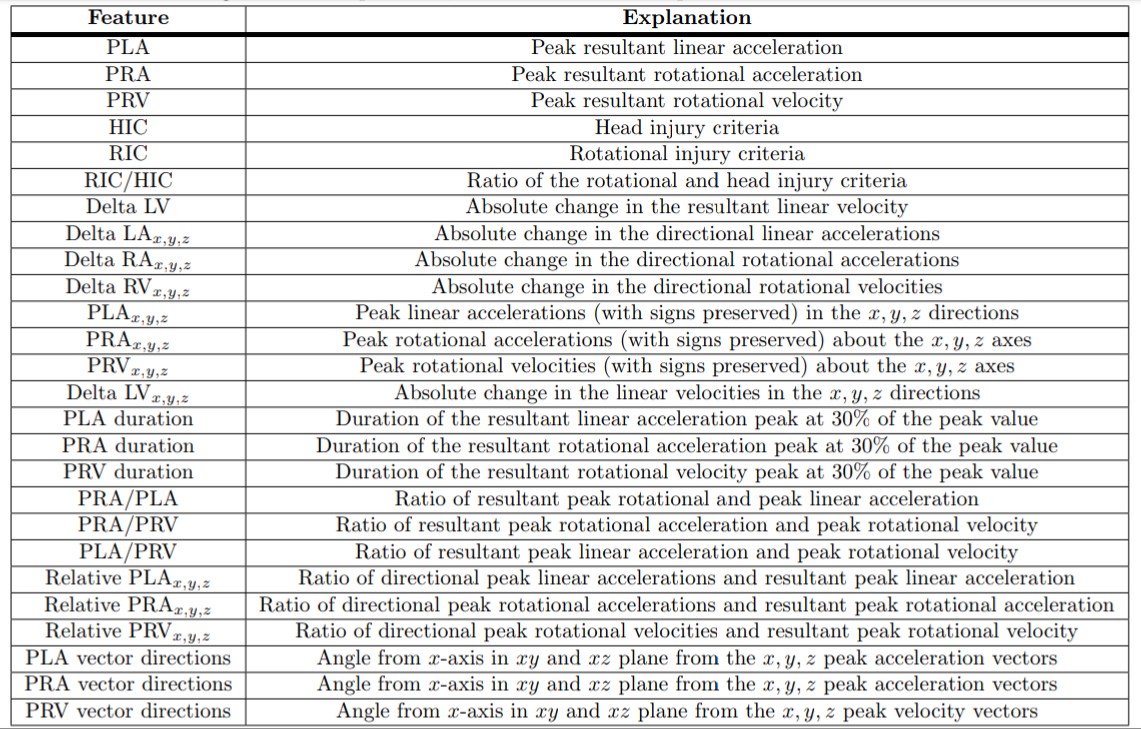

Supplement: S1 Table — (JPG) [file pone.0305986.s001.jpg]

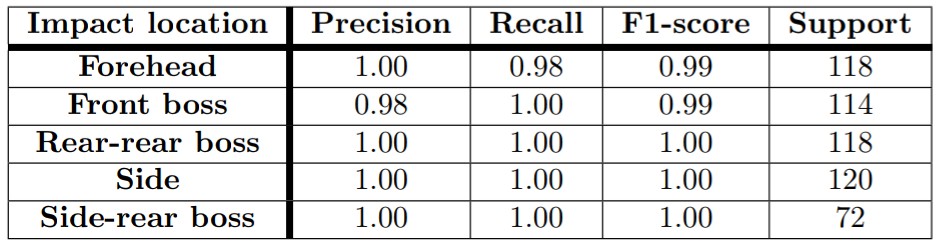

Supplement: S2 Table — (JPG) [file pone.0305986.s002.jpg]

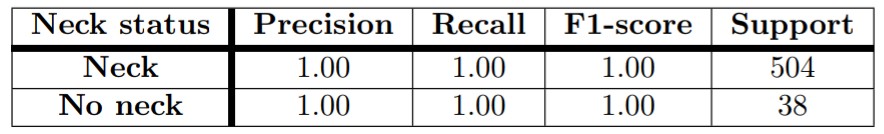

Supplement: S3 Table — (JPG) [file pone.0305986.s003.jpg]

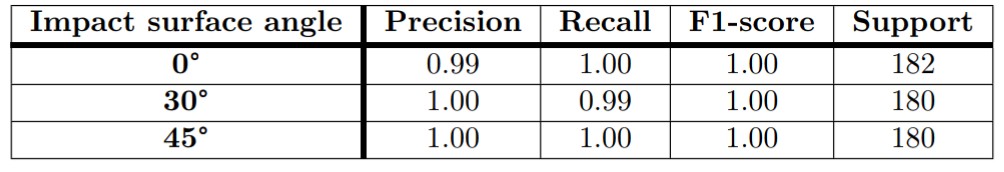

Supplement: S4 Table — (JPG) [file pone.0305986.s004.jpg]

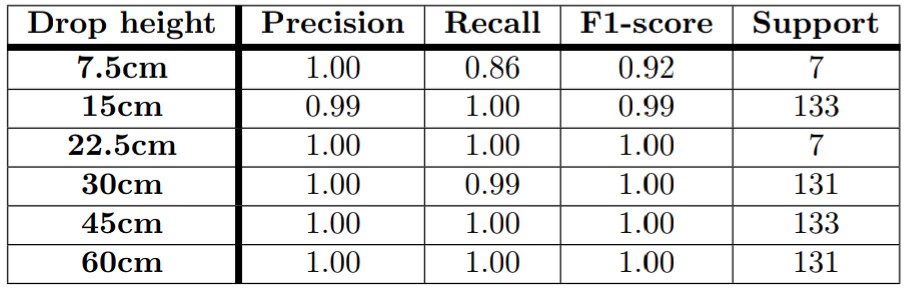

Supplement: S5 Table — (JPG) [file pone.0305986.s005.jpg]
